# Sleep quality of student athletes and non-athletes - the role of chronotype, stress and life satisfaction

**DOI:** 10.5935/1984-0063.20190153

**Published:** 2020

**Authors:** Kamila Litwic-Kaminska, Martyna Kotysko

**Affiliations:** 1 Kazimierz Wielki University, Faculty of Psychology - Bydgoszcz - Kujawsko-Pomorskie - Poland.; 2 University of Warmia and Mazury, Faculty of Social Sciences, Institute of Pedagogy, Department of Clinical, Developmental, and Educational Psychology - Olsztyn - Warminsko-Mazurskie - Poland.

**Keywords:** Sleep, Stress, Psychological, Circadian Rhythm, Students, Health

## Abstract

**Objective:**

There are many internal and external factors that can affect sleep deterioration. The adopted model of the relationship between chronotype, stress, life satisfaction and sleep quality was veriﬁed in the study.

**Material and Methods:**

In total, 335 healthy university students were surveyed using the Morningness-Eveningness Questionnaire, Perceived Stress Scale, Satisfaction with Life Scale and Pittsburgh Sleep Quality Index. The study included two groups: individuals involved in sport activities (student athletes, n=207) and those who declared (in the short form of the International Physical Activity Questionnaire) low physical activity level (non-athlete students, n=128).

**Results:**

Student athletes were less stressed (p<0.001) and declared higher life satisfaction (p<0.001) and sleep quality (p<0.001) compared to non-athletes. Non-athletes tended to identify the evening hours as their best time for functioning (p<0.001), but the mean results of both groups oscillated around the so-called intermediate type. Despite the differences in mean values, the model invariance for both groups was conﬁrmed, which means that the proposed theoretical model applies equally to student athletes and non-athletes. The path analysis results indicate that chronotype has a direct negative inﬂuence on sleep quality (preferring morning hours results in higher sleep quality). However, perceived stress partially mediates this relationship (p<0.001).

**Discussion:**

Sleep quality should not be considered without taking into account circadian preferences. Effective coping with stress may also be a buffer in reducing sleep problems.

## INTRODUCTION

### Sleep quality

The role of sleep is not without significance for athletes, for whom having good sleep quality is a desirable condition due to its role in recovery^1^ and athletic performance^2^. The results of studies performed on athletes indicate that the group deals with decreased sleep quality^3-5^.

Academic athletes, who need to combine the roles of a student and an athlete, may be more likely to suffer from sleep problems resulting from the fact that, in comparison to their peers, who are solely students, they bear more social roles. Study results, however, are ambiguous. Among the non-athlete students, the percentage indicating the occurrence of poor sleep quality is diversified and ranges from 50%^6^ to 60%^7^. Research by Mah et al.^8^ and Sheehan et al.^9^ revealed that among student athletes, more than 40% in the former study and 30% in the latter study could be classified as poor sleepers. Further, Driller et al.^10^ compared athletes and the non-athletes regarding sleep behavior and sleep quality. Generally, the first group presented poorer sleep behaviors than their non-athlete peers, but their sleep quality, measured with the Pittsburgh Sleep Quality Index (PSQI), was higher. Recent study of Arbinaga et al.^11^ demonstrated that insufficiently physically active students scored higher in the PSQI than sufficiently physically active students, and among them the number of participants who can be identified as poor sleeper is greater. The opposite results were presented by Bender et al.^12^. Their research revealed that, in comparison to a group of elite athletes, in a group of non-athletes there were more good sleepers.

### Chronotype and sleep quality

One of the issues that have been discussed due to the interconnectedness with sleep is a chronotype. Even though it is defined as a continuum between extreme morningness and extreme eveningness, particular individuals can also be classified as different chronotypes: the morning (“larks”, M-types [morning types]), the evening (“owls”, E-types [evening types]), or the neither/intermediate type (N-/I-types [neither/intermediate types])^13,14^. The M-type individuals, in contrast to the E-types, wake up and perform mentally and physically at their best in the earlier (morning) hours, but they find it difficult to stay awake at late-night hours^15,16^.

It remains unclear whether sleep quality can be explained by chronotype. Bender et al.^12^ indicate that eveningness is positively correlated with poorer sleep quality in the group of athletes (in non-athlete controls, there is no such association). In turn, Lastella et al.^17^ and Monma et al.^18^ do not indicate the effect of the chronotype on athletes’ sleep quality.

However, athletes who simultaneously hold the role of a student experience a lot of additional demands that may distort their natural circadian rhythm (e.g., early hours of lectures at the university or late hours of going to sleep due to their roommates’ late-night activities). It may lead to the so-called social jet lag, understood as both the misalignment of biological and social time^19^ and the alterations between sleep patterns on free days and workdays^20^.

### Stress and sleep quality

Prior findings prove that the difficulty with sleep can be an effect of increased stress perceived by an athlete before an important performance^5,21-23^. Juliff et al.^21^ suggest that poorer sleep quality prior to a competition constituted situational rather than a general problem with sleep. However, the results obtained by other researchers contradict this statement and indicate the existence of a negative relationship between sleep quality and general stress^18,24,25^.

Apart from numerous burdens connected with training and competitions, student athletes are also exposed to the demands of everyday life resulting from various social roles, such as a student, a friend, a partner in a relationship, or an employee^26,27^. Therefore, academic athletes may experience a higher level of stress and be particularly exposed to inappropriate sleep patterns consequently. In his review, Watson^28^ suggests that sleep restriction may not only worsen an individual’s academic and sport performance, but also increase one’s stress level, and secondarily result in further sleep disorders.

### Life satisfaction and sleep quality

The relationship between sleep quality and life satisfaction, measured within non-clinical populations, is generally positive^29,30^. Lower sleep quality is accompanied by lower satisfaction in the physical, psychological, social and environmental domains^31^. Shin and Kim^32^ demonstrated that among students sleep quality has a direct positive effect on the level of life satisfaction. They indicate, though, that the relationship between these two variables can be bidirectional. Litwic-Kaminska and Kotyśko^33^ have presented similar conclusions in a study. In this study, however, only average life satisfaction results of academic athletes who obtained scores indicating good and poor sleep quality in the PSQI were compared. The athletes with decreased sleep quality, in comparison to the good sleepers, presented a lower level of life satisfaction. It should be noted that there is a lack of research among athletes in which the relationship between sleep quality and life satisfaction is analyzed.

### Justification and objectives

Previous analyses^33^ have led us to the conclusion that student athletes with low sleep quality demonstrate a higher level of stress and declare lower life satisfaction than those with good sleep quality. The aim of this paper is to verify the relationships between the chronotype, perceived stress, life satisfaction and sleep quality. Along with this aim, we checked if stress and satisfaction with life are significant mediators in the relationship between chronotype and sleep quality.

## MATERIALS AND METHODS

### Participants

In the presented study, 349 students from one of Polish universities were surveyed. The participants consisted of two groups: student athletes (involved in sports, understood as training in sports clubs, as part of an academic sports association or having an individual training routine) and non-athletes (not engaged in sports activities and declaring a low level of physical activity). The first group consisted of 209 student athletes (aged *M* ± *SD*: 21.14 ± 1.77 yrs., 73% male). Due to the missing data in their questionnaires, two participants were excluded. The athletes represented individual (58.45%, e.g., athletics, swimming, combat sports) and team sports (41.55%, e.g., soccer, handball, volleyball, rowing), and their sports achievements were diverse. In the second group (*n*=140), the level of physical activity was screened with the usage of the short form of the International Physical Activity Questionnaire (IPAQ)^34^. Twelve students whose physical activity levels were assessed as high, due to the data they provided, were excluded. Finally, the surveyed group included 128 non-active students (aged *M* ± *SD*: 21.52 ± 2.94 yrs., 23.4% male).

### Measures

Chronotype: The Polish version of the Morningness-Eveningness Questionnaire (MEQ)^35^, prepared by Jankowski and Ciarkowska^15^, is a one-dimensional measure that allows specifying a person’s sleep-wake cycle and the preferred hours of functioning. It comprises 21 items, with usually four or five descriptive answers that are scored from one to four or five points. The higher the score, the more intense the morningness preference. The Polish adaptation of the MEQ has a satisfactory reliability level of 0.83^15^.

Perceived stress: The Perceived Stress Scale (PSS-10)^36^ is a 10-item scale that evaluates the intensity of perceived stress during the previous month. The responses are given on a five- point Likert-type scale. The total scores range from 0 (no stress) to 40 points (extreme stress). The reliability of the Polish version was α=0.86^37^.

Life satisfaction: The Satisfaction with Life Scale (SWLS)^38^ measures the cognitive component of well-being. It contains five statements (referring to one’s life), to which the examined person responds by answering on a seven-point scale. The possible range of scores is 5-35. Cronbach’s α coefficient of the version adapted to the Polish conditions is 0.81^39^.

Sleep quality: The Pittsburgh Sleep Quality Index (PSQI)^40^ assesses sleep quality over a one-month time interval. The questionnaire includes 19 items (four of them are descriptive, and the remaining 15 are weighted on a 0-3 scale) that allow distinguishing seven components that are summed to produce a global score (range from 0 to 21, where higher scores indicate lower sleep quality). Results above 5 indicate “poor sleep”^40^. In this study, the cutoff criteria for low sleep quality were set at 5 or more points^1^. The originally reported Cronbach’s α in Buysse et al.^40^ was 0.83.

Physical activity level: The Polish version of the short IPAQ^34^ contains seven questions and gathers information about the time spent sitting or walking as well as the time devoted to intensive and moderate physical activity within the previous seven days. Only the activities that lasted continuously for at least 10 minutes without any significant breaks are taken into consideration. An examined person evaluates the number of days within a week devoted to their physical activity together with an average daily duration of that activity (in hours and minutes). By meeting particular criteria determined in the total weekly activity coefficient, calculated on the basis of metabolic equivalent of work (MET), with the units expressed in MET min/week^41^, the subjects can be divided into three physical activity categories: high, moderate and low/insufficient. A short version of the IPAQ has acceptable measurement properties^42^.

### Procedure

Before the start of the activities, the participants of the study from both groups were familiarized with the aim of the research and asked to provide written informed consent for participation in the study. Following ethical principles of academic research, each participant was assured that he or she could resign from further participation at any stage of the study and that the collected data would be used for scientific purposes only. The participants individually filled out a set of questionnaires, and each received detailed feedback on the results obtained. The project of the study was approved by the Ethics Committee for Scientific Research of the Institute of Psychology (currently Faculty of Psychology), Kazimierz Wielki University in Bydgoszcz, Poland.

### Statistical analyses

Statistical analyses were performed in SPSS v25 and AMOS v25^43^. Data gathered in the study were normally distributed. The comparison between the student athletes and the physically inactive students in accordance with the PSQI, MEQ, PSS and SWLS was conducted by employing the *t*-Student test. The effect size for mean differences was interpreted using the Cohen’s guidelines. The primary analysis was hierarchical regression analysis, where it was tested if the MEQ, PSS and SWLS are significant predictors of the PSQI. The next step was path analysis (with the use of multi-group analysis), which was conducted to verify the model and to determine the direct and indirect impacts of the chronotype, stress and life satisfaction on sleep quality. The multi-group analysis makes it possible to check if the model differs depending on the group, we are analyzing (in this study - student athlete or non-athlete). In the path analysis, the maximum likelihood (ML) estimation was used to obtain model parameters. In accordance to model verification the following model fit indices were evaluated: the χ^2^, the root mean square error of approximation (RMSEA), the comparative fit index (CFI), and the standardized root mean squared residual (SRMR). Following Hu and Bentler^44^ recommendations, in order to be able to speak of a good model fit, the fit indices should assume specific values, which are as follows: RMSEA < 0.06, CFI > 0.95, SRMR < 0.08.

With regard to the χ^2^ test, a non-significant test result is an indicator of a good fit. All parameters were calculated with the bootstrap of 5.000 samples and a confidence interval of 90%.

## RESULTS

### Differences between student athletes and non-athletes

The information about the means, standard deviations, and *t*-Student test results are presented in Table 1. The significant differences between the student athletes and their inactive peers were present in all studied variables. The non-athlete students had higher scores in the MEQ than the athletes. Further, the non-athlete students demonstrated lower stress, higher life satisfaction, and better sleep quality in comparison to the athletes. The effect size of the observed differences can be identified as generally low; the difference seems to be medium only in the case of perceived stress. Using the χ^2^, test it was checked whether the numbers of participants who can be called “bad sleepers” (a score of 5 or greater in the PSQI) and “good sleepers” (a score below 5 in the PSQI) are similar when taking into account the activity level (student athletes *vs* inactive students). Among the student athletes, more participants were classified as good sleepers (*n*=123 *vs n*=44), unlike among the inactive students (χ^2^ =19.85; *p*<0.0001).

**Table 1:** Descriptive statistics for all study variables in athletes and non-athletes (n=335).

	M±SD		t	*p*	d
	Athletes	Non-athletes			
	*n* = 207	*n* = 128			
Chronotype (MEQ)	59.03±7.16	55.29±8.32	4.369	< 0.001	.48
Perceived stress (PSS)	14.73±6.08	18.01±6.40	-4.693	< 0.001	.52
Life satisfaction (SWLS)	21.98±4.65	20.09±5.25	3.447	0.001	.38
Sleep quality (PSQI)	4.40±2.24	5.38±2.27	-3.860	< 0.001	.43

### Verification of the adopted model

To determine which variables are important predictors of sleep quality, and to what extent they explain the variance of this variable, a hierarchical regression analysis was performed with the control of sex (Table 2). According to the results, it can be stated that lower scores in chronotype (β=-0.241, *p*<0.001), a higher level of perceived stress (β=0.273, *p*<0.001), and lower scores in life satisfaction (β=-0.149, *p*=0.005) were significantly associated with lower sleep quality (higher scores in the PSQI indicate lower sleep quality). Sex were an insignificant predictor of PSQI scores when all variables were included in the model.

**Table 2:** Hierarchical regression analysis of sleep quality predictors (n=335).

	β	t	*p*	R2	Δ R^2^	Δ F	*p*
**Step 1**							
Sex	0.176	-3.256	0.001	0.031	0.031	10.602	-
**Step 2**							
Sex	-0.136	-2.673	0.008	0.148	0.118	45.808	< 0.001
MEQ	-0.345	-6.768	< 0.001				
**Step 3**							
Sex	-0.069	-1.414	0.158	0.248	0.099	43.744	< 0.001
MEQ	-0.250	-4.989	< 0.001				
PSS	0.338	6.614	< 0.001				
**Step 4**							
Sex	-0.085	-1.733	0.084	0.266	0.018	8.026	0.005
MEQ	-0.241	-4.848	< 0.001				
PSS	0.273	4.902	< 0.001				
SWLS	-0.149	-2.833	0.005				

Note: Sex = 0 - female, 1 - male; MEQ = Morningness-Eveningness Questionnaire (chronotype measure); PSS = Perceived Stress Scale (stress measure); SWLS = Satisfaction With Life Scale (life satisfaction measure).

**Figure 1:**
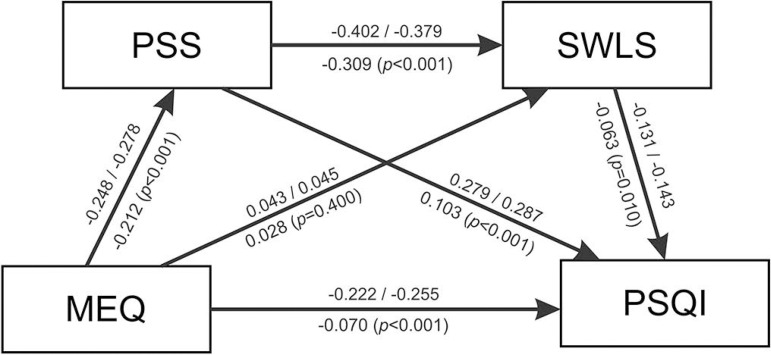
The proposed model of relationships between variables included in the study - the structural weights model. MEQ = Morningness-Eveningness Questionnaire - chronotype measure; PSS = Perceived Stress Scale - stress measure; SWLS = Satisfaction With Life Scale - life satisfaction measure; PSQI = Pittsburgh Sleep Quality Index - sleep quality measure. Values above arrows - standardized regression weights (student athletes/non-athletes) and below arrows - unstandardized regression weights.

The hierarchical regression analysis became the basis for verifying the model of specific relationships between variables in which the main outcome was sleep quality; the chronotype was treated as the main predictor and perceived stress and life satisfaction as mediators (Figure 1).

Using the multi-group analysis, we checked the fit indices of three models and compared them with the χ^2^ test. The comparison included Model 1 - unconstrained, and two constrained models (with fixed parameters); Model 2 - structural weights; and Model 3 - structural residuals (Table 3). Model 2 and Model 3 (both constrained) were not significantly worse than Model 1 (base model). Model 3 is not significantly worse than Model 2: χ^2^ =5.36, *df*=4, *p*=0.253. According to the mentioned results, we can assume that the models are equivalent, which means that the proposed theoretical model applies to the student athletes as well as to the non-athletes. In the proposed model (structural weights model was chosen), all but one path were statistically significant (Figure 1).

**Table 3:** Comparison of fit indices of three analyzed models

	Unconstrained		
	Model 1	Model 2	Model 2
		Structural weights	Structural residuals
*χ*^2^ (*df*)	0.00 (0)	3.33 (6)	8.69 (10)
*p*	-	0.766	0.562
*χ*^2^/*df*	-	0.555	0.869
GFI	1.000	0.995	0.987
CFI	1.000	1.000	1.000
RMSEA	-	0.000	0.000
		[0.000 - 0.049]	[0.000 - 0.054]
PCLOSE	-	0.954	0.932
SRMR	0.000	0.019	0.024

Based on the result of the mediation analysis, we can assume the perceived stress partially mediates the relationship between the chronotype and sleep quality (Table 4).

**Table 4:** Mediation analysis - total, direct and indirect effects (constrained model: structural weights).

	Effect value	BootLLCI	BootULCI	SE	*p*
MEQ → PSQI					
Total	-0.097	-0.124	-0.071	0.016	< 0.001
Direct	-0.070	-0.094	-0.044	0.015	< 0.001
PSS as mediator Indirect	-0.028	-0.041	-0.017	0.007	< 0.001

Note: Unstandardized effect value; Bias-corrected confidence intervals method was used to set the bootstrap confidence intervals; PSQI = Pittsburgh Sleep Quality Index (sleep quality measure); MEQ = Morningness-Eveningness Questionnaire (chronotype measure); PSS = Perceived Stress Scale (stress measure).

## DISCUSSION

The main purpose of the presented study was to examine how the chronotype, stress, life satisfaction, and sleep quality could be interrelated and whether stress and life satisfaction could modify the relationship between the chronotype and subjective sleep quality.

It was assumed that the chronotype directly affects the quality of sleep, but this relationship is mediated through the perceived stress, which results in a likely possibility of sleep quality being modified by satisfaction with life. The model parameters were tested in order to verify whether they apply to both analyzed student groups, the athletes and the non- athletes, to the same extent. Based on the obtained results, a solution was adopted assuming that the regression weights in both groups will be equal for the respective variable pairs, since the model with fixed parameters did not differ from the unconstrained one. This action allows for drawing simultaneous conclusions for both groups. In the model, all but one paths were significant. The chronotype has a negative influence on sleep quality score (but it needs to be emphasized that the higher the score, the worse the quality of sleep). This relation is in line with the findings of Roeser et al.^45^, but their study included female students only. Also, in Bender et al.^12^ research, it was noted that the athletes with the evening preference demonstrated poorer sleep quality. Yet, in the latter study, this relationship was only significant for the athletes; in the control group, it was inconsequential. A possible explanation could be that in the study by Bender et al.^12^ the control group consisted of the participants who could be classified as good sleepers; in our study, we included the students with low physical activity, and, as showed, their sleep quality was lower than the athletes’.

The chronotype significantly influences the perceived stress, as well. The presented results are consistent with the data obtained by Roeser et al.^45^, in which the chronotype had a significant impact on the level of the self-perceived stress response (this study procedure included an induction of acute stress to the participants via mental arithmetic task); however, this relationship became insignificant when sleep quality was included as a mediator. The model of relationship between the variables, developed as a part of our own study, included the reverse relationship among three variables, in comparison to the study by Roeser et al.^45^, for example, a given chronotype, sleep quality and perceived stress. We assumed that the chronotype could affect sleep quality directly as well as indirectly, via the perceived stress. This assumption was developed by the current research results indicating that stress affects the subjective quality of sleep^18,24,28^.

Our findings from the mediation analysis suggest that the perceived stress (measured by a questionnaire and related to everyday life) is a significant mediator in the mentioned relation between the chronotype and sleep quality (partial mediation), which means that perceived stress can further aggravate sleep problems that arise from the chronotype of an individual. However, it should be mentioned that the chronotype itself, as a preference for functioning within selected times of the day, does not have to be a problem. Merely a discrepancy between the chronotype and the requirements of social functioning (i.e., social jet lag) may result in subjectively lower sleep quality.

The question about the possible two-way relationship between the mutual influence of stress and the quality of sleep has remained unresolved. In our proposed model, a path leading from stress to sleep quality was set; however, it is possible that reversing the model (the direct relation of the chronotype and stress, which is mediated by sleep quality, as proposed by Roeser et al.^45^) and scrutinizing this relationship will give similar results.

Apart from the verification of the adopted model of relations between the variables, analysis of the differences between the studied groups of students was performed. The athlete students obtained higher results in the chronotype, were less stressed, and declared higher life satisfaction and sleep quality. We believe that this requires an extensive interpretation.

Primarily, note that higher scores on the MEQ indicate higher morningness preference, and conversely, lower scores indicate higher eveningness tendency. The mean results of both groups allow considering the participants as intermediate types, although the average result of the inactive students is near the limit score for E-type. Similarly, insufficiently physically active students in Arbinaga et al.^11^ study indicate a greater tendency towards the eveningness than those who presented sufficient physical activity. The obtained results in our study suggest that the athletes tend to choose earlier hours for work, learning or training. A possible explanation comes from the studies discussing physical activity as one of the health behaviors. It was confirmed that when compared to the M-types, the E-types were generally less active and spent more time sitting^46^.

Another divergence between the athletes and the non-athletes was noted regarding perceived stress and satisfaction with life. Following Demirel^47^, we presumed that the athletes would declare a higher level of stress and lower satisfaction with life. Other reports suggest that participation in a sport activity may become an additional stressor that non-athletes do not experience^48^. The differences obtained in our study stand in contrast to these findings. The explanation here may be the fact that we did not examine acute stress connected with competitions or exam session periods (the time of conducting the research was chosen to limit the possibility of such situations), but we have taken into account stress experienced in everyday life. Second, physical activity undertaken by the examined students can act as a buffer against stress and the adverse effects of it. Such an effect was presented in Wunsch et al.^49^ study conducted among academic students who proved an influence of physical activity on the level of experienced stress, well-being, and sleep quality. Physical activity has a positive effect on an individual by improving his or her mood - via the release of endorphins, among other ways^50^. Generally, in most cases athletes demonstrate better mental health when compared to non-athletes, except in the case of excessive exercise intensity, incompatible with one’s possibilities at the time, and the persistent influence of stressors that an individual cannot effectively overcome^50,51^.

Most of the athletes in our study can be considered good sleepers (the results measured with the PSQI at lower than 5 points), as opposed to the non-athletes, among which about two-thirds of the participants were qualified as bad sleepers. However, 40% of the athletes still declare problems with sleep. This indicates the significance of the undertaken subject.

The presented outcomes are consistent with the findings regarding decreased sleep quality among students in general^6,7^. Analyzing the differences between the athletes and the non- athletes, it can be stated that the physically inactive students declare lower sleep quality. The previous studies have remained inconsistent in this respect. Our results are antithetical to some prior reports suggesting that athletes presented poorer sleep behaviors than the control groups^12,52,53^, but closer to the scores according to which the non-athletes have a higher PSQI global score than the athletes^10^ or sufficiently physically active students present higher sleep quality than those insufficiently physically active^11^.

### Limitations

Although this study significantly fills the research gap in respect to the quality of sleep among academic athletes, it also has certain limitations that should be noted for carrying out future research in this field. First limitation applies to the sex ratio of the subjects. However, we controlled this variable in performed analyses, subsequent research should equate these proportions, in both the group of student athletes and the group of non-athletes, in order to analyze differences between males and females regarding psychological properties.

In this study, the PSQI was used to measure the quality of sleep, but there are other methods, for example, designed solely for athletes to describe their sleep and its properties. Our study included the measurement of the control group. Therefore, the PSQI was treated as a universal questionnaire. When planning further research including student athletes, it would be advisable to adapt to Polish conditions one of the methods addressed to athletes (e.g., The Athlete Sleep Behavior Questionnaire^54^ or the Athlete Sleep Screening Questionnaire^55^) and measure sleep quality using several tools.

## CONCLUSION

In comparison to non-athletes, athletes are less stressed and declare greater life satisfaction. Stress is a partial mediator in the relationship between chronotype and sleep quality. We believe that effective coping with stress may be a buffer to help reduce sleep problems.
